# Zwitterionic Nanocellulose-Based Membranes for Organic Dye Removal

**DOI:** 10.3390/ma12091404

**Published:** 2019-04-30

**Authors:** Carla Vilela, Catarina Moreirinha, Adelaide Almeida, Armando J. D. Silvestre, Carmen S. R. Freire

**Affiliations:** 1 Department of Chemistry, CICECO – Aveiro Institute of Materials, University of Aveiro, 3810-193 Aveiro, Portugal; catarina.fm@ua.pt (C.M.); armsil@ua.pt (A.J.D.S.); cfreire@ua.pt (C.S.R.F.); 2 Department of Biology and CESAM, University of Aveiro, 3810-193 Aveiro, Portugal; aalmeida@ua.pt

**Keywords:** bacterial nanocellulose, poly(2-methacryloyloxyethyl phosphorylcholine), zwitterionic nanocomposites, dye removal, water remediation, antibacterial activity

## Abstract

The development of efficient and environmentally-friendly nanomaterials to remove contaminants and pollutants (including harmful organic dyes) ravaging water sources is of major importance. Herein, zwitterionic nanocomposite membranes consisting of cross-linked poly(2-methacryloyloxyethyl phosphorylcholine) (PMPC) and bacterial nanocellulose (BNC) were prepared and tested as tools for water remediation. These nanocomposite membranes fabricated via the one-pot polymerization of the zwitterionic monomer, 2-methacryloyloxyethyl phosphorylcholine, within the BNC three-dimensional porous network, exhibit thermal stability up to 250 °C, good mechanical performance (Young’s modulus ≥ 430 MPa) and high water-uptake capacity (627%–912%) in different pH media. Moreover, these zwitterionic membranes reduced the bacterial concentration of both gram-positive (*Staphylococcus aureus*) and gram-negative (*Escherichia coli*) pathogenic bacteria with maxima of 4.3– and 1.8–log CFU reduction, respectively, which might be a major advantage in reducing or avoiding bacterial growth in contaminated water. The removal of two water-soluble model dyes, namely methylene blue (MB, cationic) and methyl orange (MO, anionic), from water was also assessed and the results demonstrated that both dyes were successfully removed under the studied conditions, reaching a maximum of ionic dye adsorption of *ca*. 4.4–4.5 mg g^−1^. This combination of properties provides these PMPC/BNC nanocomposites with potential for application as antibacterial bio-based adsorbent membranes for water remediation of anionic and cationic dyes.

## 1. Introduction

The need for water remediation systems designed to eliminate contaminants and pollutants ravaging water sources is a global problem and, thus, is part of the goals of the 2030 Agenda for Sustainable Development [1]. Nevertheless, the struggle to remove heavy metal ions, pesticides and other dissolved organic pollutants is a difficult war and some of the efforts of researchers to accomplish such a target are directed towards the development of environmentally friendly porous nanomaterials [2,3]. In fact, big bets are being placed on systems derived from naturally occurring polymers, such as polysaccharides [4,5]. Within the portfolio of commended biopolymers, cellulose and its nanoscale forms, namely cellulose nanocrystals (CNCs), cellulose nanofibrils (CNFs) and bacterial nanocellulose (BNC), show tremendous potential for environmental and water remediation as recently reviewed [6,7,8]. The high interest in the latter nanocellulose, *viz*. the exopolysaccharide BNC biosynthesized by some non-pathogenic bacterial strains [9], lies in its 3D structure with an ultrafine network of physically entangled cellulose nanofibers, which is responsible for its *in-situ* moldability, shape retention, inherent biodegradability, high water-holding capacity and porous structure [10,11].

BNC, its derivatives and composites [12] have already been used in the manufacture of water remediation systems for the removal of dyes [13,14], oil [15] and heavy metals [16,17]. To the best of our knowledge, the partnership between BNC and a zwitterionic polymer has never been explored for the fabrication of nanocomposites, aiming at the simultaneous removal of anionic and cationic organic dyes. Under these premises, 2-methacryloyloxyethyl phosphorylcholine (MPC) was selected as a non-toxic polymerizable monomer due to its methacrylic functional group and zwitterionic phosphorylcholine moiety, consisting of a phosphate anion and a trimethylammonium cation [18], which are prone to establish electrostatic interactions with positively and negatively charged molecules. Furthermore, the unique hydration state [18], antimicrobial, bioinert and antifouling properties of MPC polymer [19,20,21] will be a major asset in reducing or avoiding bacterial growth in contaminated water.

The present study contemplates the fabrication of nanocomposite membranes consisting of cross-linked poly(2-methacryloyloxyethyl phosphorylcholine) (PMPC) and BNC via the one-pot polymerization of the corresponding non-toxic zwitterionic monomer (*i.e*. MPC) within the BNC three-dimensional porous network. The structure, morphology, thermal stability, mechanical properties, antibacterial activity towards *Staphylococcus aureus* and *Escherichia coli*, water-uptake capacity and removal of cationic and anionic organic dyes of the resulting nanocomposites were assessed.

## 2. Materials and Methods

### 2.1. Chemicals, materials and microorganisms

2-Methacryloyloxyethyl phosphorylcholine (MPC, 97%), 2,2′-azobis(2-methylpropionamidine) dihydrochloride (AAPH, 97%), *N*,*N*′-methylenebis(acrylamide) (MBA, 99%), methylene blue (MB, dye content ≥ 82%), methyl orange (MO, dye content 85 %) and paraffin oil (puriss., 0.827–0.890 g mL^−1^ at 20 °C) were purchased from Sigma-Aldrich (Sintra, Portugal) and used as received. Ultrapure water (Type 1, 18.2 MX·cm at 25 °C) was obtained from a Simplicity^®^ Water Purification System (Merck, Darmstadt, Germany). Other chemicals and solvents were of laboratory grade.

Bacterial nanocellulose (BNC) wet membranes were biosynthesized in our laboratory using the *Gluconacetobacter sacchari* bacterial strain [22]. *Staphylococcus aureus* (ATCC 6538) and *Escherichia coli* (ATCC 25922) was provided by DSMZ – Deutsche Sammlung von Mikroorganismen und Zellkulturen GmbH (German Collection of Microorganisms and Cell Cultures).

### 2.2. Preparation of PMPC/BNC nanocomposites

Wet BNC membranes (diameter: *ca*. 70 mm) with 40% water content were placed in stoppered glass-reactors and purged with nitrogen. Simultaneously, aqueous solutions of monomer (MPC, 1:3 and 1:5 BNC/MPC weight fraction), cross-linker (MBA, 5.0% w/w relative to monomer), and radical initiator (AAPH, 1.0% w/w relative to monomer) were prepared and transferred to the glass-reactors containing the drained BNC membranes. After the complete incorporation of the corresponding solution into the BNC membrane, during 1 h in ice, the reaction mixtures were heated in an oil bath at 70 °C and left to react for 6 h. Afterwards, the nanocomposites were repeatedly washed with water, oven dried at 40 °C for 12 h, and kept in a desiccator until further use. All experiments were made in triplicate and samples of cross-linked PMPC were also prepared in the absence of BNC for comparison.

### 2.3. Characterization methods

#### 2.3.1. Thickness

A hand-held digital micrometer (Mitutoyo Corporation, Tokyo, Japan) with an accuracy of 0.001 mm was used to measure the thickness of the membranes. All measurements were randomly performed at different sites of the membranes.

#### 2.3.2. Ultraviolet-visible spectroscopy (UV–vis)

The transmittance spectra of the samples were acquired with a Shimadzu UV-1800 UV-Vis spectrophotometer (Shimadzu Corp., Kyoto, Japan) equipped with a quartz window plate with 10 mm diameter, bearing the holder in the vertical position. Spectra were recorded at room temperature (RT) in steps of 1 nm in the range 250–700 nm.

#### 2.3.3. Attenuated total reflection-Fourier transform Infrared (ATR-FTIR)

ATR-FTIR spectra were recorded with a Perkin-Elmer FT-IR System Spectrum BX spectrophotometer (Perkin-Elmer Inc., Massachusetts, USA) equipped with a single horizontal Golden Gate ATR cell, over the range of 600–4000 cm^−1^ at a resolution of 4 cm^−1^ over 32 scans.

#### 2.3.4. Solid-state carbon cross-polarization/magic-angle-spinning nuclear magnetic resonance (^13^C CP/MAS NMR)

^13^C CP/MAS NMR spectra were collected on a Bruker Avance III 400 spectrometer (Bruker Corporation, Massachusetts, USA) operating at a B0 field of 9.4 T using 9 kHz MAS with proton 90° pulse of 3 µs, time between scans of 3 s, and a contact time of 2000 µs. ^13^C chemical shifts were referenced to glycine (C=O at *δ* 176 ppm).

#### 2.3.5. X-ray diffraction (XRD)

XRD was performed on a Phillips X’pert MPD diffractometer (PANalytical, Eindhoven, Netherlands) using Cu K*α* radiation (*λ* = 1.541 Å) with a scan rate of 0.05° s^−1^. The XRD patterns were collected in reflection mode with the membranes placed on a Si wafer (negligible background signal) for mechanical support and thus avoid sample bending.

#### 2.3.6. Scanning electron microscopy (SEM) coupled with energy dispersive X-ray spectroscopy (EDS)

SEM images of the cross-section of the membranes were obtained with a HR-SEM-SE SU-70 Hitachi microscope (Hitachi High-Technologies Corporation, Tokyo, Japan) operating at 4 kV. The microscope was equipped with an EDS Bruker QUANTAX 400 detector for elemental analysis. The samples were fractured in liquid nitrogen, placed on a steel plate and coated with a carbon film prior to analysis.

#### 2.3.7. Thermogravimetric analysis (TGA)

TGA was carried out with a SETSYS Setaram TGA analyzer (SETARAM Instrumentation, Lyon France) equipped with a platinum cell. The samples were heated from RT to 800 °C at a constant rate of 10 °C min^−1^ under a nitrogen atmosphere (200 mL min^−1^).

#### 2.3.8. Tensile tests

Tensile tests were performed on a uniaxial Instron 5564 testing machine (Instron Corporation, Maryland, USA) in the traction mode at a cross-head velocity of 10 mm min^−1^ using a 500 N static load cell. The specimens were rectangular strips (50 × 10 mm^2^) previously dried at 40 °C and equilibrated at RT in a 50% relative humidity (RH) atmosphere prior to testing. All measurements were performed on five replicates and the results were expressed as the average value.

#### 2.3.9. Water-uptake capacity

The water-uptake of the nanocomposites under different pH conditions was determined via immersion of dry specimens with 10 × 10 mm^2^ in aqueous solutions of 0.01 M HCl (pH 2.1), phosphate buffer saline (pH 7.4) and 0.01 M NaOH (pH 12) at RT for 48 h. After removing the specimens out of the respective medium, the wet surfaces were dried in filter paper, and the wet weight (*W*_w_) was measured. The water-uptake is calculated by the equation: $W_{uptake}\left(\%\right)=\left(W_{w}-W_{0}\right)\times W_{0}^{-1}\times 100$, where *W*_0_ is the initial weight of the dry membrane.

### 2.4. In vitro antibacterial activity

The antibacterial activity of the nanocomposite membranes was tested against *S. aureus* and *E. coli*. The bacterial pre-inoculum cultures were grown for 24 h in tryptic soy broth (TSB) growth medium at 37 °C under shaking at 120 rpm. Before the assay, the density of the bacterial culture was adjusted to 0.5 McFarland in phosphate buffered saline (PBS) solution (pH 7.4) to obtain a bacterial concentration of 10^8^ to 10^9^ colony forming units *per* mL (CFU mL^−1^). Each membrane sample (50 × 50 mm^2^) was placed in contact with 5 mL of bacterial suspension. A bacteria cell suspension was tested as the control and BNC was tested as a blank reference. All samples were incubated at 37 °C under horizontal shaking at 120 rpm. At 24 h contact time, aliquots (100 µL) of each sample and controls were collected and the bacteria cell concentration (CFU mL^−1^) was determined by plating serial dilutions on tryptic soy agar (TSA) medium. The plates were incubated at 37 °C for 24 h. The CFU were determined on the most appropriate dilution on the agar plates. Three independent experiments were carried out and, for each, two replicates were plated. The bacteria reduction of the samples was calculated as follows: *log reduction* = *log* CFU_control_ – *log* CFU_membrane_.

### 2.5. Dye removal capacity

The dye removal capacity of the PMPC/BNC nanocomposite membranes was evaluated by immersing dry samples (20 × 20 mm^2^) in 25 mL of methyl blue (MB) and methyl orange (MO) aqueous solutions (25 mg L^−1^, pH 5.7), and stirred (200 rpm) for 12 h at RT. Then, the membranes were removed from the solution and the residual concentration of dye determined by UV–vis spectroscopy (Shimadzu UV-1800 UV-Vis spectrophotometer, Kyoto, Japan) at 655 nm for MB and 463 nm for MO. Linear calibration curves for each dye in the range 0.4–3.1 μg mL^−1^ were obtained: y=0.1919x+0.0014 (*R*^2^ = 0.9991) for MB and y=0.0881x−0.0046 (*R*^2^ = 0.9992) for MO. The dye removal amount (mg g^−1^) was calculated by: $q=\left(C_{i}-C_{t}\right)\times W^{-1}\times V$, where *C*_i_ is the initial dye concentration (mg L^−1^), *C*_t_ is the dye concentration at time *t* (h), *W* is the weight (g) of the membrane and *V* is the volume (L) of the dye solution.

Additionally, a dry sample of PMPC/BNC_2 nanocomposite (20 × 20 mm^2^) was immersed in 25 mL of paraffin oil containing 1 mL of MB and MO aqueous solutions (25 mg L^−1^).

### 2.6. Statistical analysis

Statistical significance was determined using an analysis of variance (ANOVA) and Tukey’s test (OriginPro, version 9.0.0, OriginLab Corporation, Northampton, MA, USA). Statistical significance was established at *p* < 0.05.

## 3. Results and Discussion

### 3.1. PMPC/BNC nanocomposites: preparation and characterization

**Figure 1:**
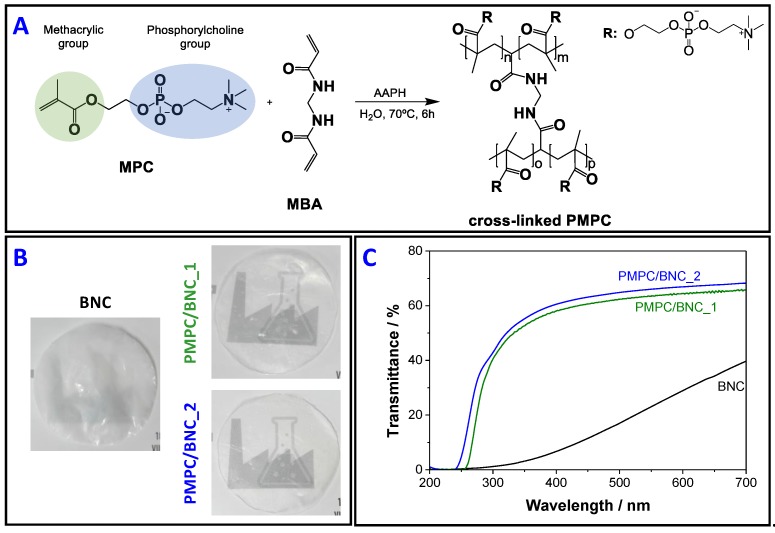
(**A**) Radical polymerization of MPC in the presence of MBA as cross-linker, yielding cross-linked PMPC, (**B**) photographs of neat BNC and nanocomposites PMPC/BNC_1 and PMPC/BNC_2, and (**C**) the corresponding UV-visible transmission spectra.

The one-pot *in-situ* free radical polymerization of MPC, inside the swollen BNC network and using MBA as cross-linker, was used to produce two PMPC/BNC nanocomposites (Figure 1) with distinct compositions (Table 1). The cross-linker was chosen based on former studies [23,24] and utilized with the goal of preserving the water-soluble zwitterionic homopolymer inside the BNC porous network during washing and utilization. The resulting nanocomposites contain 21 ± 3 wt.% and 46 ± 13 wt.% of BNC (*W*_BNC_*/W*_total_), and concomitantly 79 ± 3 wt.% and 54 ± 13 wt.% of PMPC (*W*_PMPC_*/W*_total_), which correspond to nanocomposite materials containing 479 ± 118 mg and 859 ± 90 mg of PMPC *per* cm^3^ of membrane, respectively, as listed in Table 1. The thickness of the membranes increased from 92 ± 21 µm for neat BNC to 133 ± 65 µm for PMPC/BNC_1 and 226 ± 35 µm for PMPC/BNC_2 (Table 1) due to the inclusion of the cross-linked PMPC into the three-dimensional structure of BNC. The membranes are macroscopically homogeneous with no discernible irregularities on either side of the materials surfaces, indicating a good dispersion of the cross-linked PMPC polymer inside the BNC network. After the incorporation of PMPC into the BNC network, the transparency of the nanocomposites significantly increased, as displayed in Figure 1B and confirmed by transmittance values in the visible range (400–700 nm) of 58.1–65.6% for PMPC/BNC_1 and 60.5–68.2% for PMPC/BNC_2 (Figure 1C). In the ultraviolet region (200–400 nm), the transmittance remained below 5% until 265 nm for PMPC/BNC_1 and 250 nm for PMPC/BNC_2, and then steadily increased to 58.0% and 60.5% at 400 nm for PMPC/BNC_1 and PMPC/BNC_2, respectively. Furthermore, PMPC/BNC_2 presents higher values of transmittance and concomitantly lower absorbance values, which points to a transmittance augment with higher content of cross-linked PMPC (Figure 1C). An analogous trend was observed for other BNC-based nanocomposites containing for example polycaprolactone [25], poly(methacroylcholine chloride) [23] and polyaniline [26].

**Figure 2:**
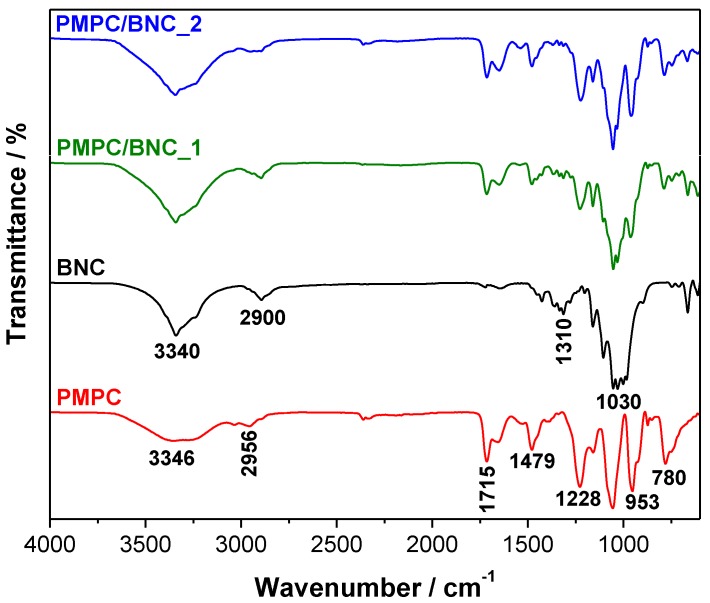
ATR-FTIR spectra of cross-linked PMPC, neat BNC, and nanocomposites PMPC/BNC_1 and PMPC/BNC_2.

The infrared spectra of neat BNC, cross-linked PMPC, and nanocomposites PMPC/BNC_1 and PMPC/BNC_2 are shown in Figure 2. The ATR-FTIR spectra of the PMPC/BNC membranes present the absorption bands of cellulose at 3340 cm^−1^ (O–H stretching), 2900 cm^−1^ (C–H stretching), 1310 cm^−1^ (O–H in plane bending) and 1030 cm^−1^ (C–O stretching) [27], jointly with those of the cross-linked PMPC at 1715 cm^−1^ (C=O stretching), 1479 cm^−1^ (N^+^(CH_3_)_3_ bending), 1228 cm^−1^ (P=O stretching), 1056 cm^−1^ (P–O–C stretching) and 953 cm^−1^ (N^+^(CH_3_)_3_ stretching) [28,29]. The presence of these absorption bands and the absence of one at about 1640 cm^−1^ corresponding to the C=C stretching of the methacrylic group of the starting monomer corroborated the occurrence of the *in-situ* free radical polymerization of MPC inside the BNC network. Furthermore, the relative intensity of the bands assigned to the cross-linked PMPC is in accordance with the *W*_PMPC_*/W*_total_ ratio measured for each nanocomposite (Table 1).

**Figure 3:**
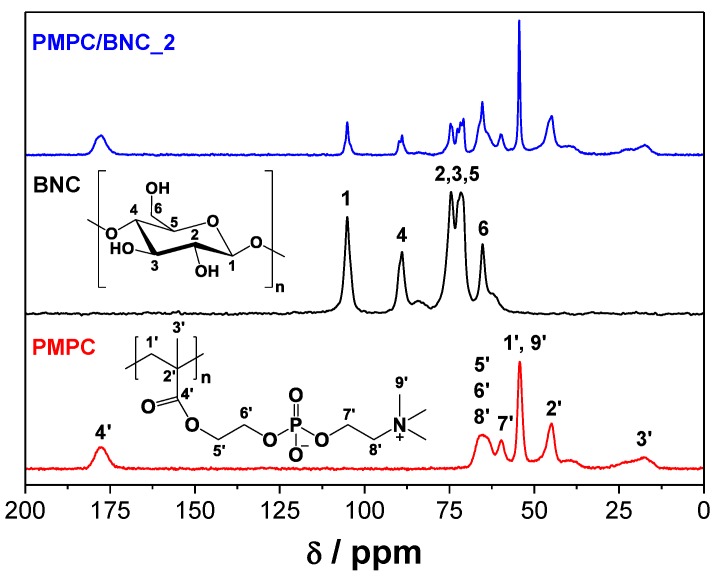
^13^C CP/MAS NMR spectra of cross-linked PMPC, neat BNC and nanocomposite PMPC/BNC_2.

The solid-state ^13^C CP/MAS NMR spectra (Figure 3) of the membranes show the typical carbon resonances of cellulose at *δ* 65.2 ppm (C6), 71.6–74.5 ppm (C2,3,5), 88.9 ppm (C4) and 105.1 ppm (C1) [27], in combination with those of cross-linked PMPC at *δ* 18.4 ppm (*C*H_3_ of polymer backbone), 44.9 ppm (quaternary C of polymer backbone), 54.3 ppm (*C*H_2_ of polymer backbone and N^+^(*C*H_3_)_3_), 59.7 ppm (O*C*H_2_CH_2_N^+^(CH_3_)_3_), 65.5 ppm (O*C*H_2_*C*H_2_O and CH_2_*C*H_2_N^+^(CH_3_)_3_) and 176.6 ppm (*C*=O). In addition, the truancy of the resonances allocated to the C=C double bond of the methacrylic group of the monomer [30] and cross-linker, supports their complete consumption during the polymerization and/or removal during the washing steps, as previously established by ATR-FTIR analysis.

**Figure 4:**
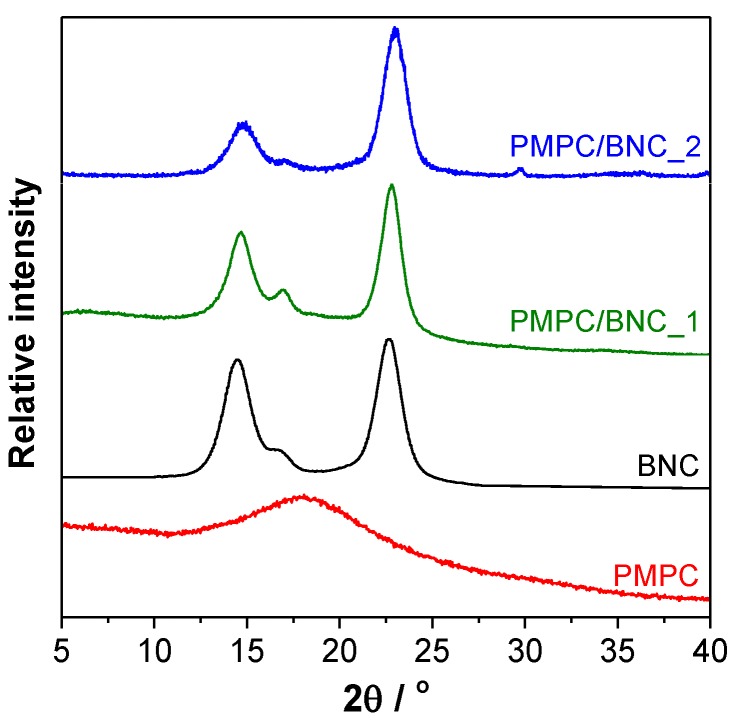
X-ray diffractograms of cross-linked PMPC, neat BNC, and nanocomposites PMPC/BNC_1 and PMPC/BNC_2.

The XRD patterns of the nanocomposites were compared with those of the individual components, namely cross-linked PMPC and BNC, to obtain an indication of the nanomaterials’ crystallinity. Figure 4 shows the amorphous character of the cross-linked PMPC with a very broad band centered at 2*θ* ≈ 18°, and the crystalline nature of BNC with a diffraction pattern characteristic of cellulose I (native cellulose) composed of highly-ordered and least-ordered regions. The nanocomposites display a diffractogram with three peaks corresponding to the (101) plane at *2θ* ≈ 14.7°, (10$\bar{1}$) plane at *2θ* ≈ 16.8° and (002) plane at *2θ* ≈ 22.8° [27], which are representative of the cellulosic substrate. The addition of the cross-linked PMPC is evident through the reduction of the peaks of the (101) and (10$\bar{1}$) planes, most likely linked to the augment of disordered cellulose domains due to the presence of the amorphous polymer. A comparable trend was reported for other BNC-based nanocomposites containing for instance poly(bis[2-(methacryloyloxy)ethyl] phosphate) [31] and poly(4-styrene sulfonic acid) [32].

**Figure 5:**
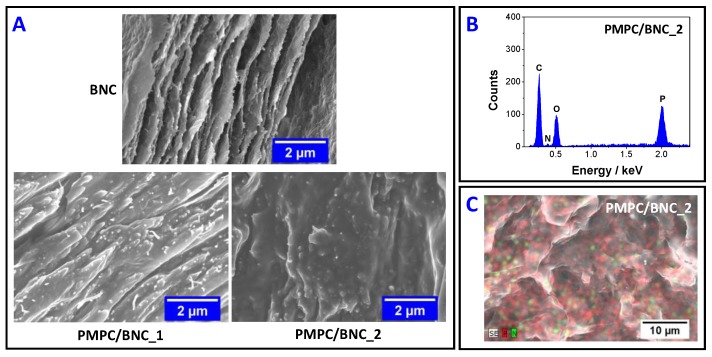
(**A**) SEM micrographs of the cross-section of neat BNC, and nanocomposites PMPC/BNC_1 and PMPC/BNC_2; EDS spectrum (**B**) and micrograph (**C**) for nitrogen and phosphorous elemental mapping of nanocomposite PMPC/BNC_2.

SEM micrographs of the cross-section of neat BNC and nanocomposites PMPC/BNC_1 and PMPC/BNC_2 are compiled in Figure 5A. It is clearly visible that the lamellar microstructure of neat BNC disappeared in the nanocomposites due to the filling of the lamellar spaces by the cross-linked PMPC, particularly in the case of PMPC/BNC_2 with 859 ± 90 mg of PMPC *per* cm^3^ of membrane. The SEM/EDS analysis reiterates the presence of PMPC and BNC through the detection of carbon (C), nitrogen (N), oxygen (O) and phosphorous (P) peaks at 0.27, 0.39, 0.51 and 2.01 keV, respectively, as illustrated in Figure 5B for PMPC/BNC_2. Moreover, the SEM/EDS mapping (Figure 5C) confirmed the uniform distribution of nitrogen and phosphorous of the cross-linked polymer within the BNC nanofibrous network, since both elements are only present in the zwitterionic PMPC.

### 3.2. Thermal stability

**Figure 6:**
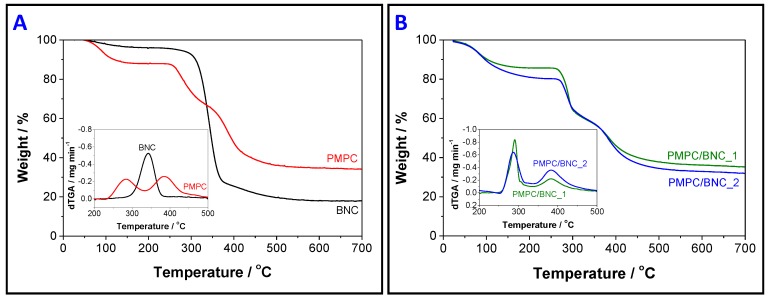
Thermograms of (**A**) cross-linked PMPC and neat BNC, and (**B**) membranes PMPC/BNC_1 and PMPC/BNC_2 under nitrogen atmosphere. The inset curves correspond to the derivative.

TGA analysis was used to study the thermal stability of the PMPC/BNC nanocomposite membranes, as well as of their individual components. Figure 6A shows the thermograms of cross-linked PMPC and neat BNC, while Figure 6B presents the thermograms of PMPC/BNC_1 and PMPC/BNC_2 nanocomposites. The thermal degradation profile of the cross-linked PMPC is characterized by two consecutive steps (apart from the dehydration at about 100 °C with a loss of *ca*. 7.5%) with maximum decomposition temperatures of 284 °C (loss of *ca*. 21%) and 385 °C (loss of *ca*. 28%, Figure 6A) corresponding to the pyrolysis of the PMPC skeleton. The thermogram of BNC shows a single weight-loss step with initial and maximum decomposition temperatures of 290 °C and 342.5 °C (loss of *ca*. 68%, Figure 6A), respectively, allocated to the pyrolysis of the cellulose skeleton [33,34]. PMPC left a residue at 800 °C corresponding to about 34% of the initial mass, whereas BNC only left a residue of *ca*. 18% at the end of the analysis.

The thermal degradation profiles of both nanocomposites (Figure 6B) follow a double weight-loss step, aside from the water loss below 100 °C (loss of *ca*. 10%). PMPB/BNC_1 has maximum decomposition temperatures at 290 °C (loss of *ca*. 24%) and 382 °C (loss of *ca*. 17%) with a final residue of 35%, while for PMPC/BNC_2 the maximum occurs at 288 °C (loss of *ca*. 19%) and 383 °C (loss of *ca*. 20%) with a residue of 32% at the end of the analysis (800 °C). This two-step pathway is associated first with the simultaneous pyrolysis of cellulose and cross-linked PMPC, and the last stage with only the zwitterionic polymer. This pattern points to the reduction of the thermal stability of the nanocomposites when compared to neat BNC, as already described for other BNC-based nanocomposites containing polymers with lower thermal stability [35]. Even so, the two PMPC/BNC membranes exhibit good thermal stability up to 250 °C (Figure 6B).

### 3.3. Mechanical properties

**Figure 7:**
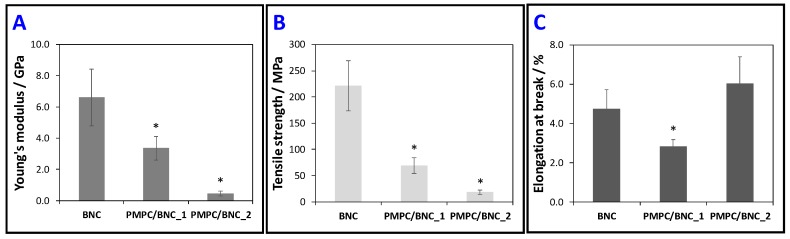
(**A**) Young’s modulus, (**B**) tensile strength and (**C**) elongation at break of neat BNC and PMPC/BNC nanocomposites; the error bars correspond to the standard deviations; the asterisk (*) denotes statistically significant differences with respect to the neat BNC (*p* < 0.05).

Figure 7 compiles the tensile tests data, namely in terms of Young's modulus, tensile strength and elongation at break, determined from the stress-strain curves. Although the tensile tests were not performed for the cross-linked PMPC due to its lack of film-forming aptitude, the cooperative effect between PMPC and BNC relies on the mechanical properties of both nanocomposites. Overall, the Young’s modulus and tensile strength of the two membranes increased with the increasing content of the cellulosic substrate (Figure 7A,B), on account of its good mechanical performance, namely Young’s modulus of 6.6 ± 1.8 GPa and tensile strength of 221 ± 48 MPa. In fact, the former parameter increased from 430 ± 150 MPa for PMPC/BNC_2 with 21 wt.% of BNC to 3.3 ± 0.8 GPa for PMPC/BNC_1 with 46 wt.% of BNC (Figure 7A), whereas the tensile strength increased from 18 ± 4 MPa for PMPC/BNC_2 to 69 ± 15 MPa for PMPC/BNC_1 (Figure 7B). In contrast, the elongation at break decreased with the increasing content of BNC from 6.0 ± 1.4% for PMPC/BNC_2 to 2.8 ± 0.3% for PMPC/BNC_1, as shown in Figure 7C. This means that the nanocomposites are more pliable than the stiff BNC nanofibers with an elongation at break of 4.7 ± 1.0%.

The dependence of the membranes’ mechanical performance on the amount of BNC is in tune with earlier studies of other BNC-based nanocomposites with polymers of low mechanical properties [14,35,36]. For example, Zhijiang et al. [14] prepared a chitosan/BNC-based hydrogel composite for dye removal, whose Young’s modulus increased from 96.5 MP for pure chitosan (dry state) to 244 MPa after the incorporation of BNC nanofibers grafted with carbon nanotubes into the chitosan hydrogel. The same behavior was obtained for the tensile strength and elongation at break [14].

### 3.4. In vitro antibacterial activity

Materials with antibacterial activity are relevant for application in multiple fields [37,38] since they can inhibit the growth and simultaneously kill pathogenic bacteria that are harmful to human health [39]. The MPC polymer is known for having antimicrobial and antifouling properties [19,20,21], which can be a major benefit in reducing/avoiding bacterial growth in contaminated water. This hypothesis was validated by assessing the growth inhibition of gram-positive (*S. aureus*) and gram-negative (*E. coli*) bacteria. *E. coli* was selected for being frequently present in contaminated water, which is a strong indication of recent sewage or fecal contamination. *S. aureus* is not so frequently present in contaminated waters; however, different strains have already been detected in urban wastewater, namely the methicillin-resistant *S. aureus* ST398 [40].

**Figure 8:**
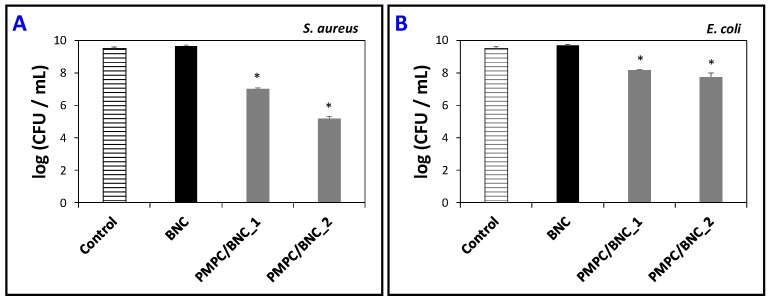
Effect of BNC, PMPC/BNC_1 and PMPC/BNC_2 on the bacterial killing (CFU) of (**A**) *S. aureus* and (**B**) *E. coli* after 24 h of exposure; error bars represent the standard deviation (three independent experiments); the asterisk (*) denotes statistically significant differences with respect to the control and neat BNC (*p* < 0.05).

Figure 8 outlines the antibacterial activity of PMPC/BNC nanocomposites and of the neat BNC membrane for comparison purposes. The inoculation of both bacteria in culture media without any sample was used as an experimental control. The neat BNC membrane, along with the experimental control, do not affect the bacterial viability of both *S. aureus* (Figure 8A) and *E. coli* (Figure 8B). This was expected given that BNC is reported not to inhibit the growth of *S. aureus* [41,42], *E. coli* [36,41,42], and other microorganisms such as *Pseudomonas aeruginosa*, *Bacillus subtilis* [42] and *Candida albicans* [43]. In fact, BNC can even be used as a substrate for microbial cell culture [44]. 

The bacterial killing of *S. aureus* by the two PMPC/BNC nanocomposite membranes is markedly concentration-dependent, as portrayed in Figure 8A. The PMPC/BNC_1 nanocomposite with 54 wt.% of cross-linked PMPC originated a significant reduction (*p* < 0.05) of bacterial concentration relatively to the control, causing a maximum of 2.5–log CFU reduction after 24 h of incubation. The PMPC/BNC_2 with 79 wt.% of cross-linked PMPC reached a higher bacterial inactivation of 4.3–log CFU reduction after 24 h, which indicates that this membrane can be considered an effective antibacterial because according to the American Society of Microbiology (ASM), every new approach has to prove an efficacy of 3–log_10_ reduction of CFU before being considered antimicrobial or antibacterial [43]. This antibacterial activity is mainly attributed to the trimethylammonium cation that is known for imparting antimicrobial properties [45]. When comparing the activity of the PMPB/BNC membranes with literature, Bertal et al. [46] verified that the triblock copolymer containing PMPC originated an inhibitory zone up to six times greater than the corresponding control against *S. aureus* and a reduction of bacterial growth by 45% compared with the experiments carried out in the absence of PMPC-based copolymer. The authors also claimed that the addition of the copolymer to a 3D-skin model infected with *S. aureus* reduced bacterial recovery by 38% compared to that of controls over 24–48 h [46].

Regarding the *E. coli* bacteria (Figure 8B), the picture is quite different and both nanocomposites exhibit a lower reduction with 1.3– and 1.8–log CFU reduction for PMPC/BNC_1 and PMPC/BNC_2, respectively. A similar behavior was reported by Fuchs et al. [47] that witnessed no antibacterial activity towards *E. coli* for one MPC copolymer. In fact, this could be expected given that *E. coli* is a gram-negative bacterium whose killing mechanism is more difficult to prevent due to the low permeability of their membranes as discussed previously in detail [48,49].

### 3.5. Water-uptake and dye removal capacity

Table 2 presents the water-uptake values for BNC and the two PMPC/BNC nanocomposite membranes after immersion in aqueous solutions of 0.01 M HCl (pH 2.1), phosphate buffer saline (pH 7.4) and 0.01 M NaOH (pH 12.0) for 48 h at RT. Overall, the water-uptake vividly increased with the increasing content of cross-linked PMPC. At pH 7.4, it increased from 101 ± 12% for neat BNC up to 639 ± 23% for PMPC/BNC_1 (54 wt.% of PMPC) and 899 ± 44% for PMPC/BNC_2 (79 wt.% of PMPC) (Table 2). In acidic aqueous solutions, PMPC/BNC_1 can absorb 6.3 ± 0.4 g of water *per* g of membrane, while for PMPC/BNC_2 the value is 9.1 ± 0.2 g of water *per* g of membrane. At pH 12, PMPC/BNC_1 absorbs 6.4 ± 0.4 g of water *per* g of membrane, whereas for PMPC/BNC_2 the water-uptake is 9.1 ± 0.3 g of water *per* g of membrane.

The larger water-uptake of the nanocomposites is correlated with the hydrophilic nature of the phosphorylcholine moiety of the cross-linked PMPC. Additionally, water-uptake is not pH-dependent since there are no significant differences (the means difference is not significant at α = 0.05) for the individual membranes under the distinct conditions of acidity or basicity. This can be explained by the unique hydration state of the PMPC chains, where the phosphorylcholine moieties have a hydrophobic hydration layer that do not disturb the hydrogen bonding between the water molecules, as discussed by Ishihara et al. [18]. This is an important characteristic in the water remediation context given that contaminated water can have different pH values. Furthermore, the higher water-uptake of PMPC/BNC_2 is an indication of a higher removal capacity of water-soluble dyes. After 48 h of immersion in aqueous solutions of different pH values, the two nanocomposites were oven dried (at 40 °C) and the final weights demonstrated that the polymer loss ranges between 1%–2%, which emphasizes the insignificant leaching of the cross-linked PMPC from the BNC network.

The removal of two model ionic organic dyes, namely methylene blue (MB) and methyl orange (MO), from water samples at room temperature after 12 h was assessed as a proof-of-concept. While MB is a heterocyclic cationic aromatic compound that is used either as a dye or a drug with for example antimalarial, antidepressant and anxiolytic activity [50], MO is a heterocyclic anionic aromatic compound that is widely used in the textile, pharmaceutical and food industries, and also as an acid-base indicator. Both azo dyes are potentially toxic towards humans and the environment [51].

**Figure 9:**
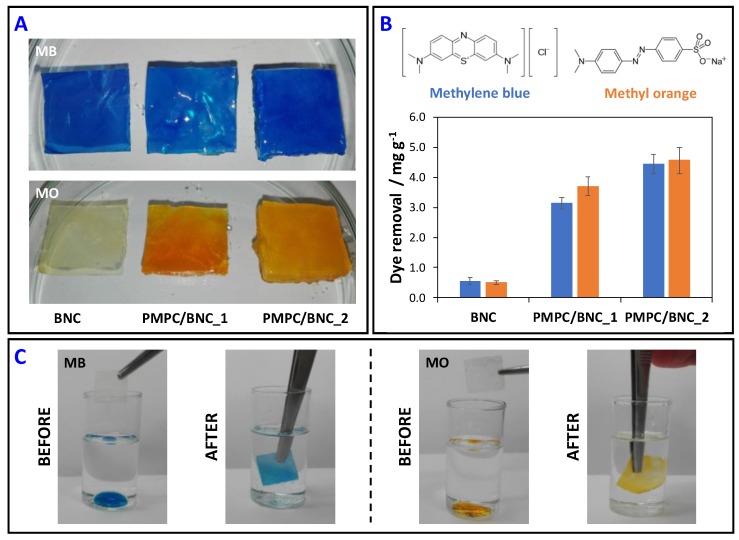
Photographs (**A**) and (**B**) dye removal capacity of BNC, PMPC/BNC_1 and PMPC/BNC_2 after 12 h of immersion in the dye aqueous solution, and (**C**) photographs of the MB and MO aqueous solutions removal from paraffin oil by PMPC/BNC_2 nanocomposite.

Figure 9A shows that the PMPC/BNC membranes can indeed retain the model water pollutants as confirmed by the different color of the nanomaterials. This is further corroborated by the data shown in Figure 9B where the dye removal capacity is plotted for each membrane. The pure BNC can remove 0.55 ± 0.12 mg of MB and 0.50 ± 0.06 mg of MO *per* g of membrane. These low removal values were expected, given the lack of binding sites in pure BNC for both cationic and anionic organic dyes. Furthermore, these values are comparable with the dyeability reported by Shim and Kim [52] in their study about the coloration of BNC fabrics with different dyes using *in situ* and *ex situ* methods.

Concerning the nanocomposites, PMPC/BNC_1 can remove 3.14 ± 0.19 mg g^−1^ of MB and 3.32 ± 0.31 mg g^−1^ of MO, whereas PMPC/BNC_2 has a removal capacity of 4.44 ± 0.32 mg g^−1^ for MB and 4.56 ± 0.43 mg g^−1^ for MO. Comparing with pure BNC, the dye removal capacity of PMPC/BNC_1 is 5.7 and 7.4 times higher for MB and MO, respectively, while PMPC/BNC_2 removes 8.1 and 9.1 times more MB and MO, respectively, than pure BNC. The higher dye removal capacity of PMPC/BNC_2 is consistent with its higher PMPC content (Table 1). Moreover, the two nanocomposites can remove both cationic and anionic dyes due to the zwitterionic nature of the cross-linked PMPC which can establish electrostatic interactions with either MB or MO model dyes. Worth mentioning is the fact that the PMPC/BNC nanocomposites can easily and quickly remove both MB and MO (25 mg mL^−1^) from the bottom of a paraffin oil container without the removal of any oil, as exemplified for PMPC/BNC_2 in Figure 9C. This is a good indication of the lack of affinity of the nanocomposites towards the hydrophobic oil and affinity for water or aqueous solutions. A similar behavior was observed for MB (aqueous solution, 100 mg L^−1^) removal from silicone oil by sulfated-cellulose nanofibrils aerogels [53].

When compared with literature, the dye removal capacity of the PMPC/BNC nanocomposites is lower than that achieved for example with highly carboxylated (COO^–^) nanocrystalline cellulose with a maximum removal capacity of 101 mg g^−1^ for MB [54], or with the amino-functionalized cellulose nanofibrils-based aerogels with 266 mg g^−1^ for MO [55]. These higher removal capacities are most likely associated with the simultaneous high content of surface binding sites and specific surface area in the first case [54], and the aerogel structure in the second case, which translates into materials with very high porosity and low density [55]. Still, the dye removal values of the PMPC/BNC membranes prepared in the present study are comparable for instance with those achieved with the sulfated-cellulose nanofibrils aerogels that removed *ca*. 5 mg g^−1^ of MB at an adsorbent dosage of 16 mg mL^−1^ [53].

Hence, the adsorbent nanocomposites developed in the present work present a customizable combination of properties, namely antibacterial activity, water-uptake and dye removal capacity, that depend on the amount of the individual components (*i.e*. PMPC and BNC), and that reveal their potential application in the context of water remediation.

## 4. Conclusions

The combination of the zwitterionic poly(2-methacryloyloxyethyl phosphorylcholine) and the hydrophilic bacterial nanocellulose yielded nanocomposite membranes that are proficient in adsorbing anionic and cationic organic dyes. The optically transparent nanocomposites display high water-uptake capacity in different pH media, thermal stability up to 250 °C, and good mechanical properties (Young’s modulus ≥ 430 MPa). Moreover, these zwitterionic membranes inhibited the growth of both Gram-positive (*S. aureus*) and Gram-negative (*E. coli*) pathogenic bacteria with maxima of 4.3– and 1.8–log CFU reduction, respectively, for the nanocomposite composed of 79 wt.% of cross-linked PMPC. Furthermore, their dye removal capacity was demonstrated by a dye adsorption amount of 4.44 ± 0.32 mg g^−1^ of MB and 4.56 ± 0.43 mg g^−1^ of MO for the membrane with the higher content of zwitterionic polymer (*i.e*. 79 wt.% of PMPC). The successful fabrication of these zwitterionic PMPC/BNC nanocomposite membranes with antibacterial activity opens novel avenues for the generation of bio-based adsorbents to address the complex issue of water remediation of anionic and cationic dyes.

## Figures and Tables

**Table 1:** List of the studied membranes with the respective weight compositions and thicknesses.

Samples	Composition ^a^			Thickness/µm
	*W*_BNC_ */W*_total_	*W*_PMPC_/*W*_total_	*W*_PMPC_/*V*_total_ (mg cm^−3^) ^b^	
BNC	1.0	–	–	92 ± 21
PMPC/BNC_1	0.46 ± 0.13	0.54 ± 0.13	479 ± 118	133 ± 65
PMPC/BNC_2	0.21 ± 0.03	0.79 ± 0.03	859 ± 90	226 ± 35

^a^ The composition was calculated by considering the weight of the nanocomposite membrane (*W*_total_), BNC (*W*_BNC_) and PMPC cross-linked polymer (*W*_PMPC_ = *W*_total_ – *W*_BNC_); ^b^ Ratio between the mass of the cross-linked PMPC (*W*_PMPC_) and the volume of the nanocomposite membrane (*V*_total_); all values are the mean of at least three replicates with the respective standard deviations.

**Table 2:** Water-uptake (water-uptake) of neat BNC and the two PMPC/BNC nanocomposite membranes at different pH media for 48 h at RT.

Membranes	Water-Uptake / %		
	pH 2.1 ^a^	pH 7.4 ^b^	pH 12.0 ^c^
BNC	109 ± 14	101 ± 12	103 ± 10
PMPC/BNC_1	627 ± 38	639 ± 23	640 ± 42
PMPC/BNC_2	911 ± 26	899 ± 44	912 ± 27

^a^ Measured after immersion in 0.01 M of HCl aqueous solution; ^b^ Measured after immersion in phosphate buffer solution; ^c^ Measured after immersion in 0.01 M of NaOH aqueous solution. All values are the mean of three replicates with the respective standard deviations.
